# ERN GENTURIS clinical practice guidelines for the diagnosis, surveillance and management of people with Birt-Hogg-Dubé syndrome

**DOI:** 10.1038/s41431-024-01671-2

**Published:** 2024-07-31

**Authors:** Marianne Geilswijk, Maurizio Genuardi, Emma R. Woodward, Katie Nightingale, Jazzmin Huber, Mia Gebauer Madsen, Dieke Liekelema - van der Heij, Ian Lisseman, Jenny Marlé-Ballangé, Cormac McCarthy, Fred H. Menko, R. Jeroen A. van Moorselaar, Elzbieta Radzikowska, Stéphane Richard, Neil Rajan, Mette Sommerlund, Maria T. A. Wetscherek, Nataliya Di Donato, Eamonn R. Maher, Joan Brunet

**Affiliations:** 1 Aarhus University Hospital, Aarhus, Denmark; 2 Dipartimento di Scienze della Vita e Sanità Pubblica, Università Cattolica del Sacro Cuore, Rome, Italy; 3 UOC Genetica Medica, Fondazione Policlinico Universitario A. Gemelli IRCCS, Rome, Italy; 4 Manchester Centre for Genomic Medicine, University of Manchester, Manchester, UK; 5 Myrovlytis Trust, BHD Foundation, Manchester, UK; 6 BHD FRANCE (a charity working closely with the BHD foundation), La Rochelle, France; 7 School of Medicine, University College Dublin, Dublin, Ireland; 8 Antoni van Leeuwenhoek Hospital, the Netherlands Cancer Institute, Amsterdam, the Netherlands; 9 Amsterdam UMC, location VUmc, Amsterdam, the Netherlands; 10 Instytut Gruzlicy Chorób Pluc, Warsaw, Poland; 11 French NCI (INCa) network for rare cancers in adults PREDIR, AP-HP, Hôpital Bicêtre, Le Kremlin-Bicêtre, France; 12 Translational and Clinical Research Institute, Newcastle University, Newcastle upon Tyne, UK; 13 Addenbrooke’s Hospital, Cambridge University Hospitals NHS Foundation Trust, Cambridge, UK; 14 Department of Human Genetics, Hannover Medical School, Hannover, Germany; 15 University of Cambridge, Cambridge, UK; 16 Aston University, Birmingham, UK; 17 Catalan Institute of Oncology, Barcelona, Spain

**Keywords:** Urological cancer, Genetic testing, Respiratory tract diseases

## Abstract

Birt-Hogg-Dubé syndrome (BHD syndrome) is an autosomal dominant multisystem disorder with variable expression due to pathogenic constitutional variants in the *FLCN* gene. Patients with BHD syndrome are predisposed to benign cutaneous fibrofolliculomas/trichodischomas, pulmonary cysts with an associated risk of spontaneous pneumothorax, and renal cell carcinoma. A requirement for updated International consensus recommendations for the diagnosis and management of BHD syndrome was identified. Based on a comprehensive literature review and expert consensus within the fields of respiratory medicine, urology, radiology, dermatology, clinical oncology and clinical genetics, updated recommendations for diagnosis, surveillance and management in BHD syndrome were developed. With the widespread availability of *FLCN* genetic testing, clinical scenarios in which a diagnosis should be considered and criteria for genetic testing were defined. Following a clinical and/or molecular diagnosis of BHD syndrome, a multidisciplinary approach to disease management is required. Regular renal cancer surveillance is recommended in adulthood and life-long, but the evidence base for additional tumour surveillance is limited and further research warranted. Recommendations for the treatment of cutaneous, pulmonary and renal manifestations are provided. Awareness of BHD syndrome needs to be raised and better knowledge of the clinical settings in which the diagnosis should be considered should enable earlier diagnosis. Further details, including areas for future research topics are available at: https://www.genturis.eu/l=eng/Guidelines-and-pathways/Clinical-practice-guidelines.html.

## Introduction

Birt Hogg Dubé (BHD) syndrome is a rare condition caused by pathogenic variants (PV) or likely pathogenic variants in the *FLCN* gene, encoding the tumour suppressor protein folliculin [1]. BHD syndrome is phenotypically heterogeneous with major manifestations in three organs: benign cutaneous fibrofolliculomas/trichodischomas (FF/TD), pulmonary cysts with an associated increased risk of spontaneous pneumothorax (PTX) and, importantly, renal tumours [2–4]. Inheritance is autosomal dominant with age-dependent penetrance and variable expression. Inter- and intra-familial phenotypic variability is common.

BHD syndrome is caused by monoallelic loss of function PVs in the *FLCN* gene [1, 3], and renal tumours are initiated by somatic mutations or loss of the wild-type allele (as in a classic tumour suppressor model) [5]. Biallelic *FLCN* inactivation leads to activation of the mTOR pathway in some contexts [6], although FLCN is implicated in additional cellular processes. A key role in the regulation of mTORC1 in kidney cells with *FLCN* inactivation is played by TFEB, that along with TFE3 belongs to the MiTF family of helix-loop-helix leucine zipper transcriptional factors [7].

The exact prevalence of BHD syndrome is unclear. A widely quoted figure is 1 in 200,000 (Source: The portal for rare diseases and orphan drugs; [8]), but the condition is generally considered to be underdiagnosed, and large-scale genomic studies of unselected clinical populations suggest that the prevalence of *FLCN* loss of function variants is around fortyfold higher [9]. However, further research is required to establish whether the risk of manifestations in individuals with pathogenic FLCN variants detected as an incidental finding is similar or lower than that in families diagnosed through clinical presentation.

Diagnosing BHD syndrome enables regular renal surveillance not only for the proband but also for relevant family members. Diagnosis may be difficult if some of the common signs, i.e. facial FF/TD, are absent or overlooked. However, a genetic diagnosis may be made even in the absence of a clear clinical phenotype.

Key clinical questions addressed in this guideline are:

- When should a potential diagnosis of BHD syndrome be considered?
- When should genetic testing of *FLCN* be considered?
- What is the optimal surveillance of target organs (lungs, kidneys, and skin) in people with BHD syndrome?
- Should specific tumour surveillance be offered to people with BHD syndrome other than for kidney cancer?
- Should any specific advice be given to people with BHD syndrome regarding the risk of pneumothorax?
- Should pneumothorax and kidney cancer in people with BHD syndrome be treated differently from those occurring in the general population?

### Scope of the guideline

The guideline applies to all individuals with BHD syndrome diagnosed on clinical findings and/or the presence of a PV in *FLCN*, and to individuals in whom a diagnosis of BHD syndrome should be considered as defined in the guideline text. This guideline is written primarily for health care clinicians who may care for patients who present with one or more of the main manifestations of BHD syndrome including clinical geneticists, urologists, dermatologists, pulmonologists, and oncologists. However, it can also be used by other physicians, patients or other interested parties. The guideline can support clinical decision making but should not replace clinical professional assessment and decision making which will be based on the individual needs, person preferences and individual circumstances of each patient. Implementation should preferably take place through the national Director of Health (or equivalent) in each European Country, but the guideline could also be disseminated through relevant medical societies including respiratory medicine, urology, oncology, radiology, dermatology and clinical genetics.

## Methods

### Evidence base and approach to secure views and preferences of target population

The guideline group for BHD syndrome was established by experts in the clinical care for the wide spectrum of manifestations of BHD syndrome and included patient representatives. The BHD Guideline Group consisted of a Core Working Group comprising ERN GENTURIS (European Reference Network for patients with a rare genetic tumour risk syndrome) clinical experts and representatives from a patient advocate group. The Core Working Group met online and drafted the guideline scope, clinical questions, recommendations and guideline document and obtained feedback from the BHD Guideline Group. The recommendations were finalised in a modified Delphi approach in which the Core Working Group, BHD Guideline Group and additional experts participated. The guideline was based on an initial review of published and indexed literature [24^th^ May 2022] that mentioned or referenced Birt–Hogg–Dubé (BHD) syndrome (total 765 papers; s*earch term - “Birt-Hogg-Dube”[All Fields])*. Other relevant papers up to August 1^st^ 2023 were also included and 71 formed the basis of the guideline. Whilst the evidence base would rate low on a formal assessment (e.g. GRADE), its strength is inclusion of case series that cover many years that appeared to have complete or near complete consecutive case reporting.

The full details of the guideline including literature search, reference list and Delphi process can be found at: https://www.genturis.eu/l=eng/Guidelines-and-pathways/Clinical-practice-guidelines.html.

## Results

### Recommendations

Using the modified Delphi process and multiple rounds of review by the BHD Guideline Group and the Core Working Group, a series of 25 recommendations were made relating to the diagnosis, management and surveillance (see Tables 1–4). Participants assessed recommendations by a four point Likert scale (totally disagree, disagree, agree, totally agree) and consensus was defined when >60% of participants responded ‘agree’ or ‘totally agree’. However, even if consensus was met, recommendations were still modified if a higher consensus was thought to be achievable from the written responses that accompanied the ratings. The strength of the recommendation was graded according to a three point scale: Strong=expert consensus AND consistent evidence; Moderate=expert consensus WITH inconsistent evidence AND/OR new evidence likely to support the recommendation; Weak=Expert majority decision WITHOUT consistent evidence.

**Table 1:** Consensus recommendations relevant to the diagnosis of BHD syndrome.

Recommendations		Strength
**Rec. 1**	A potential diagnosis of BHD syndrome should be considered* in the presence of ANY of the following:	
	a. Primary spontaneous pneumothorax.	strong
	b. Multiple bilateral pulmonary cysts, particularly in lower zone, in the absence of a known cause.	strong
	c. Bilateral or multifocal renal neoplasia (i.e. renal cell carcinomas and/or oncocytomas).	strong
	d. Renal cell carcinoma, below 50 years of age or familial.	strong
	e. Multiple cutaneous papules clinically consistent with fibrofolliculoma/trichodiscoma.	strong
	f. Any combination of the above mentioned cutaneous (e.g. multiple fibrofolliculomas/trichodiscomas), pulmonary (e.g. pulmonary cysts) or renal manifestations (e.g. renal cell carcinoma) presenting in the same individual or members of their family, with or without a known family history of BHD syndrome.	strong
	* Please note that this recommendation entails to *consider* a diagnosis of BHD syndrome, indicating that other clinical features and family history should be looked for. Recommendations to perform genetic testing to diagnose BHD syndrome can differ and are detailed in recommendation 6.	
	^ Criteria for early onset renal cell carcinoma might vary between countries and centres: specific country age recommendations for early onset renal cell carcinoma might apply.	
**Rec. 2**	A diagnosis of BHD syndrome should be considered at all ages (not just young persons) in the presence of suggestive features.	strong
**Rec. 3**	If BHD syndrome is considered as underlying diagnosis, appropriate further investigations, such as skin examination, CT scan of the lungs and/or genetic testing should be initiated.	strong
**Rec. 4**	A definitive diagnosis of BHD syndrome should be made when a genetic test is positive for a constitutive pathogenic/likely pathogenic variant in *FLCN*.	strong
**Rec. 4a**	Not all patients with clinical evidence of BHD syndrome will have a detectable *FLCN* pathogenic/likely pathogenic variant.	strong
**Rec. 4b**	A clinical diagnosis of BHD syndrome* can be made even in the absence of a detectable *FLCN* pathogenic/likely pathogenic variant if one major criterion ( >5 fibrofolliculomas or trichodiscomas, at least one histologically confirmed, of adult onset) or two minor criteria (1. Lung: bilateral basally located pulmonary cysts with no other apparent cause; 2. Kidney: early onset ( <50 years), multifocal or bilateral renal cancer, or renal cancer of mixed chromophobe and oncocytic histology; or 3. Family history: a first-degree relative with BHD syndrome) are present.	strong
	* According to the European BHD consortium criteria (Menko et al., 2009).	
**Rec. 4c**	Variants of uncertain significance (VUSs) in *FLCN* should be assessed according to international guidelines (e.g. ACMG/AMP) and interpreted in the context of the clinical presentation and familial segregation studies. Additional clinical or imaging assessments in order to detect subclinical features of BHD syndrome can also be performed.	strong
**Rec. 5**	Clinicians should be aware that BHD syndrome displays variable expression and that expecting classical features (skin lesions, pulmonary cysts and pneumothoraces) or only considering BHD syndrome in more extreme presentations (e.g. renal cell carcinoma at <40 years, pneumothorax at <40 years) might lead to the diagnosis being overlooked.	strong
**Rec. 6**	Genetic testing for *FLCN* to diagnose BHD syndrome should be a part of the genetic evaluation offered in the presence of ANY of the following:	
	a. Primary spontaneous pneumothorax if recurrent and/or familial.	strong
	b. Multiple bilateral pulmonary cysts, particularly in lower zone, in the absence of a known cause.	strong
	c. Bilateral or multifocal renal neoplasia (i.e. renal cell carcinomas and oncocytomas).	strong
	d. Familial or early onset (45 years or under)* renal cell carcinoma.	strong
	e. Multiple cutaneous papules clinically consistent with fibrofolliculoma/ trichodiscoma with at least one histologically confirmed fibrofolliculoma.	strong
	f. Any combination of these cutaneous (multiple fibrofolliculomas/trichodiscomas), pulmonary (e.g. pulmonary cysts) and renal manifestations (e.g. renal cell carcinoma) in the same individual or members of their family.	strong
	^*^ Criteria for early onset renal cell carcinoma might vary between countries and centres. From a practical perspective, specific country age recommendations for early onset RCC can be applied.	
**Rec. 7**	Predictive genetic testing for familial BHD syndrome is not generally performed until 18 years unless required for specific indications (e.g. clinical management, planning for diving activities).	strong
**Rec. 8**	First degree adult relatives of individuals with a likely pathogenic/pathogenic *FLCN* variant should be offered predictive genetic testing.	strong
**Rec. 9**	Lung ultrasound should not be used as a diagnostic test for pulmonary cysts in people with or suspected of having BHD	strong
**Rec. 9a**	A baseline low dose high-resolution computed tomography (HRCT) scan can be offered to patients with or suspected of having BHD syndrome to diagnose pulmonary cysts. This can be offered from time of diagnosis, but not usually to asymptomatic patients before 20 years of age.	moderate

**Table 2:** Consensus recommendations relevant to the clinical management of BHD syndrome.

Recommendations		Strength
**Rec. 10**	*FLCN* variants should not be considered as ‘pneumothorax-only’ variants.	strong
**Rec. 11**	All *FLCN* variants should be considered as significantly increasing renal tumour risk and lead to appropriate renal surveillance being offered.	strong
**Rec. 12**	Currently there is not sufficient evidence of an increased risk for other tumours observed in families with BHD syndrome (e.g. colorectal cancer, malignant melanoma, thyroid cancer, etc.).	moderate

**Table 3:** Consensus recommendations on surveillance in BHD syndrome.

Recommendations		Strength
**Rec. 13**	Surveillance for renal cell carcinoma should be lifelong.	strong
**Rec. 13a**	Surveillance for renal cell carcinoma should be started at age 20.	strong
**Rec. 13b**	Surveillance for renal cell carcinoma should be conducted every 1–2 years.	strong
**Rec. 14**	Surveillance for renal cell carcinoma should preferably be conducted using MRI, but ultrasound can be used if MRI is not available/appropriate.	strong
**Rec. 14a**	MRI with IV contrast should be used unless there are contraindications for contrast use.	strong
**Rec. 15**	Following the detection of a renal tumour, the frequency of imaging follow-up should increase in order to monitor growth rate and plan intervention.	strong
**Rec. 16**	Surveillance for colon polyps and/or cancers should follow local standard population or family history-based screening guidelines.	moderate
**Rec. 17**	Surveillance for thyroid cancers, salivary cancers and melanomas should not be performed as part of the routine follow-up of patients with BHD syndrome, but should be based on family history.	strong

**Table 4:** Organ-specific consensus recommendations.

Recommendations		Strength
**Rec. 18**	A formal dermatologic assessment should be considered at diagnosis.	strong
**Rec. 19**	Surgical intervention should usually be performed when the largest renal tumour reaches 3 cm in diameter.	strong
**Rec. 20**	Nephron-sparing surgery should ideally be performed whenever possible, with percutaneous thermal ablation being an alternative.	strong
**Rec. 21**	Routine Lung Function Testing is not usually required in the follow-up of asymptomatic patients with BHD syndrome.	moderate
**Rec. 22**	Risk of pneumothoraces in flying/diving should be assessed and counselled on an individual basis with specific advice from respiratory medicine based on results of high-resolution computed tomography and previous history of pneumothoraces.	strong
**Rec. 23**	Flights on commercial airlines are generally safe but for activities that may pose a risk for pneumothorax, such as working as a pilot, flying in unpressurised aircraft or diving, expert advice should be sought so that individuals can be advised appropriately.	strong
**Rec. 24**	Surgical interventions should be considered for the treatment of recurrent pneumothorax.	strong
**Rec. 25**	Ablative procedures (e.g. electrosurgery, laser therapy) to manage fibrofolliculomas and trichodiscomas (especially facial) should be considered and discussed in patients requesting intervention, particularly if a patient states their skin lesions are affecting their quality of life.	strong

The BHD syndrome guidelines are subdivided into four broad and overlapping areas: diagnostic aspects (Table 1), clinical management recommendations (Table 2), recommendations for surveillance (Table 3) and organ-specific recommendations (Table 4).

### Diagnostic aspects of BHD syndrome

The first set of Recommendations (R1 to R9a see Table 1) were related to the diagnosis of BHD syndrome. Previously various clinical diagnostic criteria for BHD syndrome have been suggested, though in general, these have been derived or adapted from those of the European BHD Consensus group (EBHDC) [4]. However since the EBHDC report there have been major changes in genetic testing which is increasingly being used as a first-line diagnostic test for BHD syndrome and access to genetic testing is no longer restricted to clinical genetics specialists. This is highly relevant to BHD syndrome as this condition may present to a wide range of clinical specialities, and it can mimic other disorders. Therefore, it is important to recognise in which clinical scenarios a diagnosis of BHD syndrome should be considered. In defining the clinical indicators of a possible diagnosis of BHD syndrome, a balance has to be made between high sensitivity/low specificity and low sensitivity/high specificity criteria in order to avoid over-investigation or under-diagnosis. Additionally, there are clinical scenarios in which the frequency of an underlying diagnosis of BHD syndrome is low, but, because of the risk of renal cell carcinoma (RCC), it is important to exclude it if possible. Recommendation 1 identified clinical presentations in which a diagnosis of BHD syndrome should be considered. Consideration of a diagnosis of BHD syndrome does not necessarily mean that genetic testing should be instigated. For example, in many countries a 49 year old man with unifocal unilateral clear cell RCC would be ineligible for routine genetic testing for hereditary kidney cancer predisposition, but the act of considering BHD syndrome might lead to the detection of a suggestive family history, lower zone cystic lung disease and/or FF/TD that then leads to genetic testing for BHD syndrome (R3). Recommendation 6 provides criteria for genetic testing for BHD syndrome.

FF/TD are the most common manifestations of BHD syndrome and show age-dependent penetrance (87 to 97% by age 70 years) [10, 11]. FF/TD appear as raised pale or skin coloured papules typically over the nose and cheeks, neck and upper trunk, and are clinically indistinguishable [12]. Their benign histopathology results in some patients not being offered treatment on this basis, however the presence of multiple facial FF/TDs can have significant psychosocial impact on people living with BHD syndrome. Less frequently FF/TD can appear on other parts of the torso and the scalp. Other skin lesions that may occur in BHD syndrome include perifollicular fibromas, comedonal FF and cystic FF. Multiple skin tags (acrochordons) are recognised in patients with BHD syndrome, though they are less specific than FF or TD as a clinical indicator [12]. Whilst multiple FF/TD are often considered pathognomonic of BHD syndrome, there are other inherited skin conditions that may mimic BHD syndrome, e.g. familial multiple discoid fibromas [13, 14]. In addition, there are two recent reports of cases with cutaneous FF and renal cancer that were associated with rare missense variants in *PRDM10* (p.Cys677Tyr and p.Cys677Arg) [15, 16]. Traditionally, skin biopsy has been performed to confirm a diagnosis of FF/TD in potential new cases of BHD syndrome, but nowadays genetic testing can offer an alternative route to diagnosis.

Approximately 10% of patients with primary pneumothoraces may have an underlying genetic cause, and BHD syndrome is the most common inherited disorder in individuals with familial pneumothorax [17, 18]. Nevertheless, the diagnosis of people with underlying BHD syndrome who first present with a pneumothorax remains an area of high unmet need. There is a longer latency to diagnosis (median 6 years) observed when pneumothorax was the first clinical feature compared to renal tumours or skin involvement [19]. There is a substantial (24–48%) cumulative lifetime risk of pneumothorax [11, 20] with at a median age at first pneumothorax of ~34 years (range 7–78 years) [19, 21]. Those who develop multiple pneumothoraces present, on average, at a younger age than those with a single occurrence (mean, 29.7 vs 38.9 years) [22]. The risk of a spontaneous pneumothorax in BHD syndrome is lifelong, so advanced age per se is not an exclusion criterion for the possibility of BHD syndrome (R2). Pneumothorax in BHD syndrome is almost invariably associated with the presence of pulmonary cysts. However, multiple pulmonary cysts are often present in BHD syndrome in individuals without a history of pneumothorax (about a third of individuals with cysts have not had a pneumothorax) [19]. An association between pneumothorax occurrence in BHD syndrome and the total number of lung cysts, total lung cyst volume and largest cyst diameter was reported [17]. BHD syndrome-associated pulmonary cysts tend to be located in the basilar regions of the lungs, in contrast to emphysematous bullae which typically occur in the upper lobes [23, 24].

The major renal manifestation of BHD syndrome is RCC which has a lifetime risk of 15–30% [10]. The earliest reported age onset of RCC in BHD syndrome is a single case at age 14 years [25], but otherwise RCC occurs after age 20 years with a median age of diagnosis of 46 years [26–28]. Predisposition to renal cancer appears to be lifelong, with RCC being diagnosed as late as 83 years [28]. Presentation with bilateral/multicentric renal cancers is well-recognised and patients who present with a single RCC may develop another primary renal tumour during follow up [28, 29].

Tumour histopathology may be an indicator of underlying BHD syndrome. Initially most RCC in patients with BHD syndrome was classified as having overlapping features of an oncocytoma and chromophobe RCC (“hybrid oncocytic/chromophobe RCC”) [26], but as the condition has been more widely recognised, other histologies have been reported e.g. chromophobe, papillary and clear cell [26, 28]. Renal oncocytoma (a benign tumour) may also occur. The age threshold for offering testing to apparently sporadic non-syndromic cases of RCC varies between health care systems (e.g. 40–50 years) [30, 31]. In addition to patients with early-onset RCC, those with multicentric or familial RCC may be routinely tested for FLCN PV as part of a panel of inherited RCC genes, individuals with BHD syndrome and RCC outside of these groups may not be tested unless other indicators of BHD syndrome are detected. In recommendation 6 the variability in age cut-offs for genetic testing “in early-onset cases” was acknowledged and a cut-off for testing of 45 years or less was suggested. However, it was also suggested that consideration of a diagnosis of BHD syndrome should extend up to age 50 years (R1) as identification of additional features of BHD syndrome (see above and below) could then lead to genetic testing being offered.

*FLCN* is the major gene associated with the BHD syndrome, though recent reports suggest that a BHD syndrome-like phenotype can rarely be associated with specific missense PV in *PRDM10* (see above). Genetic testing for *FLCN* variants may be performed as a targeted single gene test or be a part of a multigene panel or exome/genome sequencing. In practice, most testing is performed as part of a multigene panel for indications such as multicentric RCC or familial pneumothorax. The vast majority of PV are truncating variants (premature stop-codon, frameshift, canonical splice site variants) detected by DNA sequencing [8, 32]. Recurrent pathogenic variants (e.g. frameshift c.1285delC/dupC) have been described. The overall sensitivity of the genomic sequencing to detect SNVs/indels within the coding region of FLCN should be up to 100%. Individuals who are negative by sequencing may have an undetected FLCN structural variant, an intronic/noncoding variant or a PV in PRDM10 [33, 34] (R4b). The reported detection rate of FLCN PV in individuals diagnosed with BHD syndrome is estimated to be 88–96% [8, 27]. The interpretation of the clinical relevance of missense variants, as well as intronic and non-coding variants in FLCN remains challenging. Only a handful of the FLCN missense variants have undergone functional characterization supporting their pathogenicity [35], and the vast majority of the identified missense variants are reported as variants of unknown significance (VUSs) (ClinVar database access March 2023: 787 missense variants, 688 uncertain; see https://www.ncbi.nlm.nih.gov/clinvar/). The classification of a variant as a VUS should be made according to standard international guidelines (e.g., ACMG/AMP) in a certified diagnostic laboratory and interpreted in the context of the clinical presentation and family segregation studies (R4c). Additional clinical or imaging assessments to detect subclinical features of BHD syndrome can also be performed to aid variant interpretation (R3).

Following the detection of a *FLCN* PV, at risk family members (e.g. first degree relatives or second degree if intervening relative is unavailable) can be offered cascade testing to enable those who test negative to be released from regular surveillance (R8). In general, as for other adult-onset hereditary tumour predisposition syndromes, predictive testing is not performed before the age of 18 years unless the results would influence the management of the at risk child (R7). [36, 37].

### Clinical Management of BHD syndrome (Tables 1, 2)

Following the diagnosis of BHD syndrome in an individual, whether by clinical criteria (R4b) or, more commonly, by the detection of a constitutional pathogenic *FLCN* variant (R4a), the focus switches towards ongoing management of any current BHD syndrome-related complications and surveillance to reduce the morbidity from complications that might develop in the future. This is exemplified by measures to ensure that any renal tumours are detected at an early stage (<3 cm diameter). Reports of a constitutional pathogenic *FLCN* variant in kindreds with a “familial pneumothorax only” phenotype led to suggestions that BHD syndrome and isolated familial pneumothorax might be allelic [38]. However, a systematic review of the literature observed that cases of *FLCN*-related familial pneumothorax only were, on average, younger and from smaller families than individuals reported with additional manifestations of BHD syndrome. It was concluded that all individuals with a constitutional *FLCN* PV, even in the presence of a personal and family history of a pneumothorax only phenotype, should be considered to be at risk of renal tumours and offered appropriate renal surveillance (R10, R11) [19]. In addition, no other putative genotype-phenotype correlations have been confirmed to date.

Individuals with a clinical diagnosis of BHD syndrome (see R4b, Diagnostic aspects of BHD syndrome) but no detectable *FLCN* PV should be managed in a similar manner as those with a detectable PV. There is little information on the management of PRDM10-associated BHD-like syndrome. For the time being caution should be used as applying an approach similar to patients with classical BHD syndrome might not be sufficient, particularly if there is a history of aggressive RCC in which case the “3 cm rule” for renal tumours (see below) may not be applied [16]. Further data is needed to determine how such PRDM10-associated BHD-like syndrome cases should be managed.

### Aspects of surveillance in BHD syndrome (Table 3)

As with other cancer predisposition syndromes, a major focus for the ongoing care of individuals with BHD syndrome is the early detection of BHD syndrome-related neoplasia. Risks calculated to age 70 of developing a RCC in BHD syndrome have been estimated as 15–30% and, as in other hereditary cancer syndromes, renal tumour surveillance is offered in BHD syndrome [39]. CT and MRI scans are more sensitive than ultrasonography for detecting small renal masses, but MRI scans avoid the radiation loads associated with annual CT scans [40]. Though some centres employ ultrasonography for renal surveillance in BHD syndrome, additional data is required to define if the reduced sensitivity for detecting small renal lesions compared to MRI will lead to the underdiagnosis of clinically significant RCC (R14, R14a**)** [41]. Surveillance usually commences at age 20 years [2, 4, 39] as RCC rarely occurs earlier [25]. In the absence of another life-impairing illness, and if agreed with the patient, RCC surveillance may continue for life (R13, R13a), since RCC has been reported in the ninth decade in BHD syndrome patients [28]. It is recommended that renal imaging is performed every 1–2 years, preferably annually. Further research is required to determine if more detailed imaging (MRI) might enable intervals to be extended (R13b). When a renal tumour is detected, the frequency of imaging follow-up should be increased to monitor growth rate and plan intervention (see later) (R15).

Whilst it has been suggested that BHD syndrome may predispose to a variety of other neoplasms, including colorectal, thyroid and salivary gland tumours, and melanoma, to date, none of these possible associations have been confirmed sufficiently to indicate that specific surveillance is required (R12) [3, 20, 42–49]. The most investigated potential association has been with colorectal neoplasia. Evidence for [42] and against [20, 44] an association between BHD syndrome and colorectal neoplasia has been reported and currently a risk for colorectal cancer is considered unproven (though there could be an increased risk of colorectal polyposis) [44]. This has led to suggestions that standard population screening guidelines for colorectal cancer should be used in BHD syndrome [50], and, when there is a positive family history of colorectal cancer, then case surveillance recommendations should be individualized according to local guidance for familial colorectal cancer (R16). Similarly, though it has been recommended that a formal dermatologic assessment should be conducted at diagnosis (R18), there is no evidence to suggest that ongoing surveillance for melanoma (and other non-renal tumours) is indicated in BHD syndrome (R17) [50].

### Organ-specific management recommendations (Table 4)

#### Skin

Self-reported alterations in Health-Related Quality of Life (HRQOL) was reported in approximately one-third of patients with BHD syndrome and FF/TD [51], and, though routine dermatology clinic surveillance is not required, a formal assessment should be considered at diagnosis (R18), with rereferral as required. Therapeutic management is available with standard dermatological approaches, including shave excison, punch excision, ablative electrosurgery [52] and laser therapy, and should be considered as an effective intervention for substantially improving the Quality of Life (R25). Treatment is not curative and may need to be repeated. Topical rapamycin (mTOR pathway inhibitor) has been tested in a single trial and was found not to be useful as a treatment for established FF/TD [53].

#### Lung

A low dose high-resolution computed tomographic (HRCT) of the chest may be performed at diagnosis (R9a) [50, 54]. The number and size of cysts may indicate pneumothorax risk and so inform personal counselling [17, 55]. However, there is no evidence supporting regular repeated imaging of the chest and it should only be repeated when clinically indicated [50]. Pulmonary cysts are best defined by HRCT scans and lung ultrasound should not be used (R9) [56]. Little has been published regarding the impact of BHD syndrome on lung function. In a retrospective study Daccord et al. [57] assessed clinical data regarding 96 individuals and found that BHD syndrome-related cystic lung disease did not affect respiratory function at baseline, except for slightly increased residual volume and reduced carbon monoxide transfer factor (DLCO) [57]. No significant syndrome-specific deterioration of lung function was seen in the limited follow-up period of 6 years [57]. Currently, the consensus is that routine lung function testing is not indicated in asymptomatic individuals with BHD syndrome (R21). Data regarding pneumothorax risk during air travel and diving is limited, although it has been estimated that BHD syndrome patients have a pneumothorax risk of 0.63% per flight and a risk of 0.33% per episode of diving [58]. In accordance, Gupta et al. (2017) found a similar low occurrence of pneumothorax during flying and also that the risk decreases in patients who have undergone pleurodesis [54]. Anecdotally, the risk of pneumothorax may be higher in unpressurised aircraft. Individuals with BHD syndrome who plan to work as a pilot or dive regularly should seek specialised advice regarding the risks and potential preventative interventions [59] (R22, R23). Treatment of pneumothorax in BHD syndrome does not differ from that of pneumothorax for other reasons. Surgical intervention, (e.g. Video Assisted Thoracoscopic Surgery (VATS) and chemical or mechanical pleurodesis or pleurectomy, or total pleural covering) should be considered in case of recurrent pneumothorax [60] (R24, R25).

#### Kidney

If renal tumours are identified, they should be followed with interval imaging studies until the largest tumour diameter reaches 3 cm, at which point nephron-sparing intervention should be pursued R19. This ‘3 cm rule’ was originally formulated in von Hippel-Lindau disease, but has been widely adapted for several other hereditary renal cancer predisposition syndromes [2, 61, 62]. An alternative to surgery is image-guided percutaneous ablative therapies such as radiofrequency ablation and cryoablation [63]. No studies have directly compared the use of partial nephrectomy and thermal ablation in BHD syndrome-related renal tumours, and it has been suggested that thermal ablation may complicate the interpretation of post-treatment imaging surveillance and surgical procedures in patients at high risk of new tumours [62]. However, recent studies have reported that percutaneous thermal ablation can be applied successfully for the treatment of renal tumours in people with BHD syndrome [64, 65], and, within the limited evidence available, appears to be safe and effective (R20). Further studies assessing the safety of thermal ablation in BHD syndrome-related renal cancer are needed.

## Discussion

This ERN GENTURIS guideline on BHD syndrome covering aspects of the diagnosis, genetic counselling, surveillance, and clinical management of BHD syndrome was developed from the best available evidence and the consensus of experts with input from patients and a patient advocate group. The ERN GENTURIS guidelines have similiarities to previous reports on the management of BHD syndrome. The clinical diagnostic criteria for BHD syndrome suggested by the EBHDC [4] were adopted both for the current consensus (R4b) and others [60] though a specification that the diagnostic criteria for lung cysts should include development before 40 years of age has been reported [2]. Annual MRI starting at age 20 years for renal tumour surveillance is similar to the EBHDC recommendation [4] though longer scanning intervals have been mentioned by some groups e.g. “at least every 36 months” for MRI scans [50, 62, 66]. The “3 cm rule” with nephron-sparing intervention for the management of renal tumours in BHD syndrome has also been broadly agreed [2, 4, 39, 62, 66]. Sriram et al. have suggested that a chest CT scan should be performed on all patients with a primary spontaneous pneumothorax and that genetic testing for *FLCN* PV should then be considered in those found to have multiple pulmonary cysts [67]. Gupta et al. concluded that patients with BHD syndrome should be reassured that BHD syndrome-associated cystic lung disease typically does not result in respiratory failure but patients with pulmonary impairment should be followed up by a pulmonary physician with periodic assessment of pulmonary function. They also recommended evaluation by a lung specialist prior to air travel if there was evidence of pulmonary impairment or extensive cystic lung disease or prior pneumothorax and that patients should not travel when suffering from unexplained chest pain or dyspnoea [20]. Schmidt et al. noted that the World Recreational Scuba Training Council had recommended that a history of spontaneous pneumothorax should be a contraindication to scuba diving (even if following pleurodesis) [2]. In addition, it has been recommended that smoking should be discouraged in all cases [60].

BHD syndrome is a multisystem disorder and it is important that a named clinician should take responsibility for ensuring the overall coordination of surveillance and clinical management. A diagnosis of BHD syndrome may be associated with psychological challenges and socioeconomic hardships, though the occurence of these differs between individuals. Potential psychological effects can include anxiety related to uncertainty about future health problems and/or fear of developing cancer. Coping with a chronic health condition may trigger or exacerbate depression and, particularly when facial FF are numerous, there may be concerns about body image with self consciousness about physical appearance leading to social withdrawal and depression. BHD syndrome, like other inherited disorders, might impact on family relationships and dynamics. Affected parents may be anxious about their untested children and feel guilty if their children become affected. Among siblings, unaffected relatives may feel guilty if their sibling is affected. Couples may feel stress and anxiety when making plans for starting a family and there may be emotional distress if there are differing perceptions of the implications of having an affected child and a lack of consensus over their reproductive options. Therefore addressing the psychological needs of patients and families with BHD syndrome should form a key element of holistic health care for BHD syndrome. Clinicians should be sensitive to indicators of anxiety, depression, emotional distress etc, enquire about wellbeing at each clinical contact and organise appropriate referral for professional support as required. Peer-to-peer support through patient support groups can also play a key role in maintaining wellbeing.

As with many other rare diseases, a limitation to the development of consensus guidelines was the lack of research evidence for different areas of BHD syndrome management (e.g. the psychological consequences of BHD syndrome and for approaches to ameliorate them). The formulation of these guidelines for the diagnosis and management of BHD syndrome inevitably highlighted those areas in which further research is required. These are described in the full guideline document, which is available on the ERN GENTURIS website. Although these guidelines will inevitably require revision in the light of future new evidence, the current recommendations have high levels of agreement from clinical experts and patient representatives and should enhance the care of individuals affected by, or at risk of, BHD syndrome.
